# Epidemiological situation of acquired immunodeficiency syndrome (AIDS)-related mortality in a municipality in northeastern Brazil. A retrospective cross-sectional study

**DOI:** 10.1590/1516-3180.2017.0130100917

**Published:** 2018-01-15

**Authors:** Luana Rodrigues da Silva, Ellen Thallita Hill Araújo, Moisés Lopes Carvalho, Camila Aparecida Pinheiro Landim Almeida, Adélia Dalva da Silva Oliveira, Patrícia Maria Gomes de Carvalho, Tatyanne Silva Rodrigues, Viriato Campelo

**Affiliations:** I RN. Nurse, Undergraduate Nursing Department, Centro Universitário UNINOVAFAPI, Teresina (PI), Brazil.; II RN. Master’s Student, Department of Business Sciences, Fundação Sousândrade, Universidade Atlântica, São Luís (MA), Brazil.; III RN. Doctoral Student, Department of Biochemistry and Photodynamic Therapy Applied to Biomedical Engineering, Universidade do Vale do Paraíba (UNIVAP), São José dos Campos (SP), Brazil.; IV RN, MSc, PhD. Titular Professor, Master’s Program on Family Health, Centro Universitário UNINOVAFAPI, Teresina (PI), Brazil; V RN, MSc, PhD. Coordinator, Undergraduate Nursing Department, Centro Universitário UNINOVAFAPI, Teresina (PI), Brazil.; VI RN, MSc, PhD. Coordinator, Undergraduate Nursing Department, Universidade Federal do Piauí (UFPI), Teresina (PI), Brazil.; VII RN. Master’s Student, Postgraduate Program on Nursing, Universidade Federal do Piauí (UFPI), Teresina (PI), Brazil.; VIII MD, MSc, PhD. Coordinator, Master’s Program on Science and Health, Universidade Federal do Piauí (UFPI), and Physician at the Court of Justice of the State of Piauí (TJ/PI), Teresina (PI), Brazil.

**Keywords:** Epidemiology, Mortality, Acquired immunodeficiency syndrome, Health information systems, Brazil

## Abstract

**CONTEXT AND OBJECTIVE:**

The number of acquired immunodeficiency syndrome (AIDS)-related deaths covers different segments of the population differently, making monitoring of this mortality essential. The aim of this study was to describe the epidemiological situation of AIDS-related mortality in a municipality in the northeastern region of Brazil.

**DESIGN AND SETTING:**

Retrospective cross-sectional study based on data from death certificates in the mortality information system of the Health Information Center, Municipal Health Foundation, Brazil.

**METHODS:**

Between 2003 and 2013, we investigated death certificates on which AIDS-related mortality was reported. Sociodemographic data, year, place, type of establishment where death occurred and underlying and associated causes that led to AIDS-related death were described. The Mann-Kendall test was used to verify the growth trend of the standardized mortality rate over the period studied.

**RESULTS:**

Among the 1,066 AIDS-related deaths, 69.7% were among men; 47.2% of the individuals were 28-41 years of age, 32.7% had had 4-7 years of schooling, 66.9% were *pardos* (mixed race), 55.7% were unmarried and 15.3% were housekeepers. Hospitals were the site of 97% of the deaths, and 91% occurred at public hospitals. Respiratory failure was the main cause of death. The prevalence of infectious and parasitic diseases was 99.0%. AIDS-related mortality increased by 160% over the period studied, from 5.5/100,000 inhabitants in 2003 to 14.3/100,000 in 2013.

**CONCLUSION:**

In the Brazilian municipality studied here, AIDS-related mortality was most prevalent among men and young adults of lower socioeconomic level. Over the period studied, the mortality rate increased.

## INTRODUCTION

The high number of deaths due to acquired immunodeficiency syndrome (AIDS) in different segments of the Brazilian population has been studied within several fields of science. It is very important to gain knowledge of the underlying circumstances and causes of this infectious disease. However, its high mortality rate represents a constant challenge within healthcare professionals’ practice and for the healthcare system. Such challenges may reflect institutional, professional and personal changes.

AIDS is an infectious disease caused by the human immunodeficiency virus (HIV), which affects the immune system and gives rise to immunosuppression, with T-CD4+ lymphocyte deficiency and dysfunction and impairment of the cellular immune response.^1^ This infection has a large social dimension because of the large number of people infected with HIV. By the end of 2015, worldwide, 38.8 million people were living with HIV.^2^ In 2010, Brazil had 630,000 people with the virus, which resulted in 34,500 new AIDS infections each year. Among the virus carriers, 255,000 were unaware that they were carrying HIV.^3^^,^^4^

A United Nations report on the worldwide situation showed that, in 2001, there were about 3.4 million new cases of HIV infection. In 2012, about 35.3 million people were living with HIV, with a significant increase in the number of cases of infection. At the same time, the number of AIDS-related deaths was declining, such that there were 1.2 million deaths in 2014.^3^

According to data from the Brazilian Ministry of Health’s Department of Sexually Transmissible Diseases (STDs), AIDS and Viral Hepatitis, the state of Piauí, located in the northeastern region, had an AIDS mortality coefficient of 2.4/100,000 inhabitants (2007-2013), which was lower than the AIDS-related mortality rate for Brazil (5.6/100,000 inhabitants). These data suggest that AIDS epidemiology and mortality span different segments of the population in different manners. Thus, epidemiological studies have emphasized the importance of caring for HIV/AIDS patients through provision of antiretroviral drugs at healthcare services.^5^

Universal access to antiretroviral therapy became available in Brazil in 1991 through use of zidovudine monotherapy (AZT). In 1996, highly active antiretroviral therapy was introduced with a combination of three drugs. By 2006, more than 16 drugs were available for treating AIDS in Brazil.^6^ Antiretroviral therapy is started when the levels of defense cells (T-CD4+ lymphocytes) are below 350 cells/mm³, or when patients have some symptoms, with or without opportunistic infections.^7^

Because of the difficulty of regularly obtaining comprehensive, reliable and comparable mortality data, the Ministry of Health implemented a national epidemiological surveillance system and a single death certificate (DC) model in 1975. These measures resulted in establishment of the Mortality Information System (SIM/MS), which is responsible for compiling data on deaths that occur throughout the national territory, thus enabling construction of demographic and health indicators for the population.^8^^,^^9^

In this context, the present research was justified by the high number of AIDS-related deaths in different segments of the population and the importance of determining the circumstances and the underlying and associated causes. Moreover, given the lack of scientific studies on the epidemiological situation of AIDS-related mortality in the northeastern region of Brazil and, more specifically, in the municipality of Teresina, state of Piauí, monitoring of these deaths becomes essential.

## OBJECTIVE

The objective of this study was to describe the epidemiological situation of AIDS-related mortality in the municipality of Teresina, located in the northeastern region of Brazil, using unified data from death certificates stored in the Mortality Information System.

## METHODS

This was a cross-sectional, quantitative, descriptive and retrospective study. It used data on mortality due to AIDS in the municipality of Teresina that was available in the SIM. The information was gathered at the Health Information Center of the Municipal Health Foundation (NUINSA-FMS) in the city of Teresina, Brazil. There was good data coverage and satisfactory data quality.

Teresina, the capital of the state of Piauí, is in the central-western part of the state and the mid-northern part of the northeastern region of Brazil. It has an area of approximately 1,756 km². The population was 814,230 (380,612 men, 40.6%, and 433,618 women, 59.4% in 2010), with 767,557 people living in the urban area and 46,673 inhabitants in the rural area.^10^

The inclusion criteria for selecting the cases were that they needed to be AIDS-related deaths reported in the SIM between 2003 and 2013, which occurred in the city of Teresina. Data collection took place in September 2014, using a form constructed according to data contained in death certificates, which are also available in the SIM. The study variables were sociodemographic characteristics (gender, age, schooling, marital status, race/color and occupation) location and type of establishment (public or private institution) where deaths occurred, year of death and causes that led to death from AIDS.

The results were organized through insertion of data into the Excel software. The data were presented in the form of absolute numbers (N), relative frequencies (%) and graphs containing information on sociodemographic characterization and description of the underlying causes of death from AIDS. Mortality coefficients were constructed using the number of AIDS-related deaths in the municipality of Teresina and the year, and these data were obtained from the SIM/MS. The specific coefficients of AIDS-related mortality per 100,000 inhabitants were calculated considering the population living in Teresina, Brazil. All data collected were processed in the Statistical Package for the Social Sciences (SPSS) software, version 21.0. The Mann-Kendall test was used to ascertain the growth trend of the standardized mortality rate over the period studied.

This project was implemented after approval was received from the Research Ethics Committee of the UNINOVAFAPI University Center, Teresina, Piauí, Brazil, through ethics approval certificate (CAAE) no. 34750114.5.0000.5210 and report no. 771 803, in August 2014.

## RESULTS

**Figure 1:**
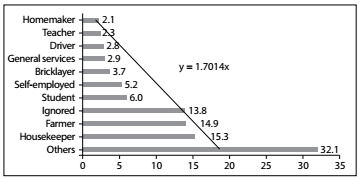
Occupations of the individuals who died due to the acquired immunodeficiency syndrome (AIDS) in Teresina (PI), Brazil, 2003 to 2013 (n = 1066).

Among the 1,066 deaths due to AIDS in Teresina between 2003 and 2013, the highest numbers of cases occurred among men (69.7%) and among people aged 28-41 (47.2%). Although the high rate of unknown data (21%) hampered the analysis on schooling, the majority (32.7%) of the people for whom this was recorded had attended school for 4 to 7 years (complete elementary schooling). In relation to marital status, there was a higher prevalence of unmarried people (55.7%). The race or color informed by the relatives was *pardo* (mixed) in 66.9% of the cases (Table 1). Figure 1 shows the distribution of deaths according to the type of occupation or activity performed.

**Table 1:** Sociodemographic characterization of the epidemiological situation of acquired immunodeficiency syndrome (AIDS)-related mortality in Teresina (PI), Brazil, 2003 to 2013 (n = 1066)

Variable	n	%
Gender		
Male	744	69.7
Female	308	29.0
Unknown	14	1.3
**Age group (years)**		
0-13	23	2.2
14-27	152	14.3
28-41	504	47.2
42-55	293	27.5
56-69	76	7.1
70 and over	6	0.6
Unknown	12	1.1
**Schooling (years)**		
None (illiterate)	72	6.7
1-3 (incomplete elementary education)	207	19.4
4-7 (completed elementary education)	350	32.7
8-11 (completed high school)	173	16.2
12 or more (tertiary education)	43	4.0
Unknown	221	21.0
**Marital status**		
Unmarried	583	55.7
Married	280	26.3
Widowed	50	4.8
Judicially separated/divorced	37	3.5
Unknown	116	9.7
**Race/color**		
White	111	10.4
Black	129	12.1
Asian	1	0.1
*Pardo* (mixed)	714	66.9
Indigenous	2	0.2
Unknown	109	10.3

Source of data: Health Information Center of the Municipal Health Foundation (NUINSA-FMS), Teresina (PI), Brazil.

Regarding the place where the AIDS-related deaths occurred, among the 1,066 deaths analyzed, 1,034 (97%) occurred at hospitals, 23 (2.2%) at home, six (0.6%) in an unknown place, two in other places (0.2%) and one (0.1%) at healthcare facilities other than hospitals (0.1%). Regarding the type of establishment where the deaths occurred, public institutions were most prevalent, with 970 (91%) of the deaths.

Table 2 shows the distribution of the underlying causes of AIDS-related deaths reported to the SIM over the period studied. Respiratory failure (35.6%) was the largest cause of death. AIDS was in fifth place, with 5.2% of the deaths.

**Table 2:** Description of the underlying causes of death among acquired immunodeficiency syndrome (AIDS)-related deaths reported in Teresina (PI), Brazil, 2003 to 2013 (n = 1066)

Underlying cause of death	n	%
Respiratory insufficiency	380	35.6
Cardiorespiratory arrest	121	11.4
Multiple organ failure	94	8.8
Acute respiratory insufficiency	69	6.5
AIDS	55	5.2
Septic shock	35	3.3
Sepsis	35	3.3
Neurotoxoplasmosis	31	2.9
Pneumonia	28	2.6
Pneumocystosis	24	2.3
Hypovolemic shock	12	1.1
Others	182	17.1

Source of data: Health Information Center of the Municipal Health Foundation (NUINSA-FMS), Teresina (PI), Brazil.

Regarding the causes associated with AIDS-related mortality in Teresina during the period studied, the distribution of the deaths according to the chapters of the Tenth Edition of the International Classification of Diseases and Causes of Death (ICD-10) was as follows (considering that more than one associated cause could be informed for the same death): for 1,030 deaths (99.0%), the associated causes were in Chapter I, relating to infectious and parasitic diseases; for 271 deaths (26.1%), the causes were in Chapter X, respiratory tract; for 42 deaths (4%), the causes were in Chapter XI, digestive tract; and for 35 deaths (3.4%), the causes were in Chapter IV, endocrine, nutritional and metabolic diseases. Other, less frequently associated causes were diseases of the genitourinary system (Chapter XIV), for 32 deaths (3.1%); diseases of the nervous system (Chapter VI), for 30 deaths (2.9%); and diseases of the circulatory system (Chapter IX), for 21 deaths (2.0%).

**Figure 2:**
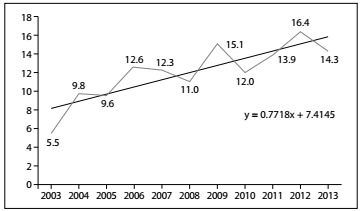
Standardized crude mortality rate for AIDS-related mortality in Teresina (PI), Brazil, 2003 to 2013 (n = 1066)

Regarding the AIDS-related mortality rate, there was an increase of 160% over the period studied, from 5.5 deaths/100,000 inhabitants in 2003 to 14.3/100,000 in 2013. Over the years analyzed, three times stood out: the year 2012, with the greatest significance, followed by 2009 and 2013. The series showed a progressive increase in mortality rates, as demonstrated by the Mann-Kendall test result (P = 0.032) and by the trend line, which confirmed that this rate will tend to increase over the coming years (Figure 2).

## DISCUSSION

The profile of AIDS-related deaths in Teresina over the period studied corroborated the findings from previous studies, which reported that the population with HIV/AIDS consisted predominantly of adult men with no stable partner who had not completed high school.^11^

According to the Ministry of Health, the coefficient of AIDS-related mortality among men is higher than among women: 8.4 and 4.2 deaths per 100,000 inhabitants, respectively.^12^ Another study confirmed that there was higher mortality among men than among women, with general ratios of 2.5 for the coefficients and 2.4 for the number of deaths. Likewise, that study showed that the population studied consisted predominantly of men (69.7%).^13^ This differed from more recent studies that have reported that the AIDS epidemic and mortality have become feminized.^14^^,^^15^

Women account for almost half of the 40.3 million people who have died due to HIV or AIDS worldwide. In Brazil, the numbers of AIDS cases among women have been increasing, in comparison with the number among men. Thus, the mortality rate due to AIDS among men has shown a more significant decrease than the rate among women.^16^

In relation to age group, the mortality rate among older adults and elderly people aged 56 to 69 years was 7.1%), while among elderly people aged 70 years and over, it was 0.6%. This demonstrates that deaths due to AIDS among elderly people were significantly frequent. A previous study carried out in Teresina, which investigated the epidemiological characteristics of the incidence of elderly people with AIDS, identified higher prevalence among elderly people aged between 60 and 69 years (88.5%).^17^

Other studies have considered schooling to be one of the main socioeconomic indicators negatively correlated with increased AIDS incidence and mortality, i.e. lower schooling would be linked to higher incidence and mortality. In this respect, the category of schooling with the highest number of deaths in the present study was that of 4 to 7 years of education (incomplete elementary school), which was concordant with other reports.^2^^-^^18^

Since the beginning of the 1990s, cases of AIDS-related mortality have become more frequent among individuals with lower educational levels. AIDS-related mortality in the years 2001 and 2010 occurred more among individuals with 4 to 7 years of schooling, thus confirming the results from the present study.^19^ One explanation for this observation is that the greater that individuals’ education level is, the greater their knowledge about AIDS will be, which provides them with better social resources to live with the disease.^20^

One important issue that needs to be addressed is the large number of records with no information about schooling (21% of the 1,066 deaths examined here). Information that is missing from death certificates is a problem faced by the epidemiological surveillance system. The main underreporting problem is that information is incomplete, especially regarding sociodemographic information, which restricts wider use of death certificates.

Concerning marital situation, AIDS-related mortality was most prevalent among unmarried people (55.7%). Research on the epidemiological profile of AIDS in Caruaru, state of Pernambuco, also reported that AIDS-related mortality was most prevalent among unmarried people (69.2%), followed by deaths among those who were married (21.2%), separated (3.8%) and widowed (2.9%). One of the theories for elucidating the high AIDS mortality rate among unmarried people is that they have a greater number of sexual partners and more frequently have unprotected sex and, thus, have greater chances of contracting the infection.^21^ With regard to married women, there is evidence showing that they face difficulty in asking their husband to use a condom, since these women are expected to trust him and not raise suspicion about his fidelity.^22^

In the present population, the largest racial group was *pardos* (66.9%), followed by blacks (12.1%) and whites (10.4%). These results differed from those of an analysis on race/color, on data in the health information systems of the Brazilian National Health System (Sistema Único de Saúde, SUS) for the city of São Paulo. According to that analysis, the distribution of deaths in this regard was stable, such that white race/color accounted for around 70.5% of AIDS-related deaths in 2010 in the city of São Paulo, while blacks accounted for 23.1% of these deaths in the same year.^23^

An epidemiological study conducted in the state of Santa Catarina showed that most patients who died from AIDS were white, followed by blacks.^24^ In a study conducted by the Ministry of Health, individuals with white skin color accounted for 52.1% of AIDS cases in Brazil, while 36.9% were *pardo* and 10.3% were black.^9^ In comparing the studies, there was a difference that could be explained by the predominance of white individuals who were the descendants of European immigrants in Santa Catarina.

Another variable analyzed was occupation, for which, like schooling, there was also a significant proportion of unknown data (13.8%). The results highlighted highest prevalences of housekeepers (15.3%) and farmers (14%). People living with HIV/AIDS who are housekeepers may have abandoned their jobs to hide their condition, while continuing to work at home, taking care of spouses and children.^25^

The result relating to the place of death shows that, among the establishments included in the survey, hospitals had the highest death rate (97% of the cases). This response is in line with previously published studies, which have reported that in most cases, people with HIV/AIDS only seek healthcare institutions after developing some type of opportunistic disease.^26^^,^^27^ In addition, public healthcare institutions predominated, accounting for 91% of deaths. These numbers reflect the change in the epidemiological situation regarding AIDS that has taken place: individuals of high socioeconomic level characterized the beginning of the epidemic; subsequently, the disease spread, such that it now mainly affects those with low income.^11^

The results showed that AIDS was the main underlying cause of death in only 5.2% of the cases. This trend was also observed in a study conducted in the state of Amazonas on 129 AIDS patients who died and were subjected to necropsy. In that study, the most frequent cause of death was tuberculosis, accounting for 28%.^28^ Use of the concept of the underlying cause of death is essential for analysis on historical trends, for comparisons between countries and for common use for standardized procedures to guide prevention of death.^29^ Respiratory insufficiency was seen to be highly prevalent among the individuals assessed in this study, affecting a total of 35.6%. A study carried out in the state of Ceará revealed that respiratory failure was the main complication (56%) among patients with AIDS, and it was a fundamental factor leading to the outcome of death, even during hospitalization (32.8%).^30^

Analysis on the distribution of deaths through the codes of causes described in the International Classification of Diseases, 10^th^ revision (ICD-10), made it possible to identify a higher rate of infectious and parasitic diseases. The group of respiratory diseases was the second largest group in importance as associated causes of death. Another study in Teresina also showed that infectious and parasitic diseases were the main causes associated with deaths AIDS-related deaths, in this city, followed by pulmonary diseases.^31^

A study carried out in São Paulo showed that AIDS was the cause of 65.3% of the deaths investigated. Among these, tuberculosis was mentioned as the main associated cause; the progression of the disease was worsened by tuberculosis, which doubled the risk of death.^32^ Another study carried out in the state of São Paulo on AIDS-related deaths that occurred in that state observed that the respiratory diseases group was in second place among the associated causes of death.^13^ Regarding pulmonary infections due to AIDS, a third study pointed out that 85% of the patients died with a low T-CD4+ count, and that pneumonia was the main associated cause.^33^

With regard to the AIDS-related mortality rate, the analysis of the present study showed that, over the period from 2003 to 2013, in Teresina, the number of deaths increased by 160%. In the first year surveyed, the rate was equivalent to 5.5 deaths/100,000 inhabitants, whereas it was 14.3 in the last year. Despite the implementation of specific healthcare policies for individuals living with HIV/AIDS and use of antiretroviral therapy, this study showed that there is a trend towards growing numbers of cases of AIDS-related deaths in Teresina.

A study carried out at a referral hospital for transmitted diseases in Piauí, located in Teresina, revealed that the numbers of reports of AIDS in municipalities with less than 50,000 inhabitants are increasing. This indicates that the mortality rate will be increasing.^17^ Brazilian research bodies have been encouraging development of further scientific studies on the epidemiological characteristics of individuals living with infectious diseases,^34^ with the objective of increasing the visibility of the Brazilian public healthcare system for investing in prevention and control strategies.

Through the results presented and discussed in this study, it is possible to consider that AIDS-related mortality has reached heterogeneously different segments of the population studied, and that even with implementation of antiretroviral therapy, the mortality rate due to the disease is still increasing. Knowledge of the epidemiological situation of individuals who died due to AIDS-related conditions is necessary for planning and evaluating healthcare. In this regard, the findings from the present study not only provide support for elaboration of new healthcare policies but also make it possible to improve the quality of the care given to these individuals and furnish scientific support for future studies.

The limitations of this study were its cross-sectional design and its use of information from death certificates obtained from public databases, considering that under-registration of information exists, as observed for the variables of gender, age, schooling, marital status and race/color. However, in view of the magnitude of the topic of this research, obtaining the results from other sources was not possible.

## CONCLUSION

AIDS-related mortality in the Brazilian municipality studied here was most prevalent among men and among young adults of lower socioeconomic level. Furthermore, the mortality rate increased over the period studied. In this light, this study may constitute a basis for elaborating new healthcare policies and for improving the quality of the care provided to these individuals. One suggestion would be to focus investments on infection prevention and control strategies and to elaborate new studies in order to broaden the scientific knowledge relating to this subject.

## References

[1] Carpes JS, Lobo E, Pereira J (2013). Morbidade e Mortalidade por HIV/Aids em Florianópolis: desafio para a gestão. Coleção Gestão Saúde Pública.

[2] Wang H, Wolock TM, GBD 2015 HIV Collaborators (2016). Estimates of global, regional, and national incidence, prevalence, and mortality of HIV, 1980-2015: the Global Burden of Disease Study 2015. Lancet HIV.

[3] (2015). On the Fast-Track to end AIDS by 2030: Focus on location and population.

[4] Santos EM, Reis AC, Westman S, Alves RG (2010). Avaliação do grau de implantação do programa de controle da transmissão vertical do HIV em maternidades do "Projeto Nascer" [Implementation evaluation of Brazil's national vertical HIV transmission control program in maternity clinics participating in the "Nascer" Project]. Epidemiol Serv Saúde.

[5] Costa Oliveira MT (2007). O diagnóstico tardio e óbito por aids de pacientes internados em 2005 em um hospital de referência para doenças infecciosas em Belo Horizonte, Minas Gerais.

[6] Lorscheider JÁ, Geronimo K, Colacite J (2012). Estudo da adesão à terapia antirretroviral para HIV/Aids de pacientes atendidos no município de Toledo/PR. Acta Biomedica Brasiliensia.

[7] Brasil. Ministério da Saúde. Secretaria de Vigilância em Saúde. Departamento de DST, Aids e Hepatites Virais (2013). Protocolo clínico e diretrizes terapêuticas para manejo da infecção pelo HIV em adultos.

[B8] Brasil. Ministério da Saúde. Secretaria de Vigilância em Saúde. Departamento de Vigilância Epidemiológica (2009). Guia de vigilância epidemiológica.

[9] Brasil. Ministério da Saúde (2009). A experiência brasileira em sistemas de informação em saúde.

[10] Secretaria do Estado da Saúde do Piauí (2012). QualiSUS Rede. Subprojeto Estadual/PI: Região de Saúde Entre Rios/Piauí.

[11] Lima RR, Santos MJL, Lira MCC, Mangueira SO, Damásio SLC (2015). Perfil epidemiológico da infecção por HIV/AIDS relacionado a atividade ocupacional [Epidemiological profile of HIV/Aids infection related to occupational activity]. Journal of Nursing UFPE On Line.

[12] Brasil. Ministério da Saúde. Programa Nacional de DST e Aids (2006). Boletim epidemiológico Aids.

[13] Santo AH, Pinheiro CE, Jordani MS (2000). Causas básicas e associadas de morte por AIDS, Estado de São Paulo, Brasil, 1998 [AIDS as underlying and associated causes of death, State of S. Paulo, Brazil, 1998]. Rev Saúde Pública.

[14] Pedrosa NL, Paiva SS, Almeida RLF (2015). Série histórica da AIDS no Estado do Ceará, Brasil [The historic data series on AIDS in the state of Ceará, Brazil]. Ciênc Saúde Coletiva.

[15] Almeida ANS, Silveira LC, Silva MRF, Araújo MAM, Guimarães TA (2010). Produção de subjetividade e sexualidade em mulheres vivendo com o HIV/Aids: uma produção sociopoética [Subjectivity and Sexuality Production in Women Living With HIV/Aids: a Sociopoetic Production]. Rev Latino-Am Enfermagem.

[16] Costa R, Silva RRA (2013). Fatores relacionados à feminização da epidemia da AIDS: estudo informativo [Factors related to feminization of the epidemy of AIDS: an information study]. Journal of Nursing UFPE On Line.

[17] Silva HR, Medeiros MOC, Figueiredo TS, Figueiredo MLF (2011). Características clínico-epidemiológicas de pacientes idosos com aids em hospital de referência, Teresina-PI, 1996 a 2009 [Clinical and epidemiological characteristics of elderly patients with AIDS in a reference hospital, Teresina-PI, 1996 to 2009]. Epidemiol Serv Saúde.

[18] Silva RAR, Duarte FHS, Nelson ARC, Holanda JRR (2013). Epidemia da Aids no Brasil: Análise do perfil atual. Journal of Nursing UFPE On Line.

[19] Faqueti A, Rodriguez AMM, Woerner CB, Antonio GD (2014). Perfil epidemiológico de mortalidade por aids na população adulta do Brasil de 2001 a 2010. Revista de Saúde Pública de Santa Catarina.

[20] Reis RK, Santos CB, Dantas RAS, Gir E (2011). Qualidade de vida, aspectos sociodemográficos e de sexualidade de pessoas vivendo com HIV/AIDS [Quality of life, sociodemographic factors and sexuality of people living with HIV/AIDS]. Texto Contexto Enferm.

[21] Maciel SSSV, Maciel WV, Andrade MC (2010). Perfil epidemiológico da Aids no município de Caruaru-PE [Epidemiological profile of aids in Caruaru City, PE]. Journal of Nursing UFPE On Line.

[22] Silva CM, Vargens OMC (2009). A percepção de mulheres quanto à vulnerabilidade feminina para contrair DST/HIV [Women's perception about female vulnerability to STD and HIV]. Rev Esc Enferm USP.

[B23] São Paulo. Secretaria da Saúde. Coordenação de Epidemiologia e Informação (2011). Análise do quesito raça/cor a partir de sistemas de informação da Saúde do SUS [Analysis of race/color from the information systems of the Brazilian health system].

[24] Schuelter-Trevisol F, Pucci P, Justino AZ, Pucci N, Silva ACB (2013). Perfil epidemiológico dos pacientes com HIV atendidos no sul do Estado de Santa Catarina, Brasil, em 2010 [Epidemiological profile of HIV patients in the southern region of Santa Catarina State in 2010]. Epidemiol Serv Saúde.

[25] Bastiani JAN, Padilha MICS, Vieira M, Maliska ICA, Maia ARCR (2012). Pessoas que vivem com HIV/AIDS em Florianópolis/SC, Brasil: ocupação e status socioeconômico ocupacional (1986-2006) [Persons living with HIV/AIDS in Florianópolis/SC, Brazil: occupation and occupational socioeconomic status (1986-2006)]. Rev Eletr Enf.

[26] Thuler LCS, Hatherly AL, Góes PN, Silva JRA (1998). Infecção pelo HIV: descritores de mortalidade em pacientes hospitalizados [Mortality descriptors in HIV inpatients]. Rev Saúde Pública.

[27] Rezende ELLF (2012). Mortalidade por aids no Brasil.

[28] Souza SLS, Feitoza PVS, Araújo JR, Andrade RV, Ferreira LCL (2008). Causas de óbito em pacientes com síndrome da imunodeficiência adquirida, necropsiados na Fundação de Medicina Tropical do Amazonas [Causes of death among patients with acquired immunodeficiency syndrome autopsied at the Tropical Medicine Foundation of Amazonas]. Rev Soc Bras Med Trop.

[29] Santo AH (2007). Potencial epidemiológico da utilização das causas múltiplas de morte por meio de suas menções nas declarações de óbito, Brasil, 2003 [Epidemiological potential of multiple-cause-of-death data listed on death certificates, Brazil, 2003]. Rev Panam Salud Pública.

[30] Pontes LB, Leitão TMJS, Lima GG, Gerhard ES, Fernandes TA (2010). Características clínico-evolutivas de 134 pacientes com histoplasmose disseminada associada a SIDA no Estado do Ceará [Clinical and evolutionary characteristics of 134 patients with disseminated histoplasmosis associated with AIDS in the State of Ceará]. Rev Soc Bras Med Trop.

[31] Campelo V, Gonçalves MAG, Donadi EA (2005). Mortalidade por doenças infecciosas e parasitárias no município de Teresina-PI (Brasil), 1971-2000 [Mortality due to infectious and parasitic diseases in the city of Teresina-PI (Brazil), 1971-2000]. Rev Bras Epidemiol.

[32] Santo AH, Pinheiro CE, Jordani MS (2003). Causas múltiplas de morte relacionadas à tuberculose no Estado de São Paulo, 1998 [Multiple-causes-of-death related to tuberculosis in the State of São Paulo, Brazil, 1998]. Rev Saúde Pública.

[33] Costa CH (2010). Infecções pulmonares na Aids. Revista Hospital Universitário Pedro Ernesto.

[34] Ferraz ML, Yoradjian A, Barbieri A (1998). Epidemiology of acute hepatitis B in a university hospital in São Paulo, Brazil: retrospective study of two five-year periods. Sao Paulo Med J.

